# Surface functionalization of TiO_2_ nanotubes with minocycline and its in vitro biological effects on Schwann cells

**DOI:** 10.1186/s12938-018-0520-6

**Published:** 2018-06-20

**Authors:** Lan A, Wenzhou Xu, Jinghui Zhao, Chunyan Li, Manlin Qi, Xue Li, Lin Wang, Yanmin Zhou

**Affiliations:** 1 Department of Oral Implantology, School and Hospital of Stomatology, Jilin University, Changchun, 130021 China; 2 Key Laboratory of Tooth Development and Bone Remodeling in Jilin Province, Changchun, 130021 China; 3 Department of Periodontology, School and Hospital of Stomatology, Jilin University, Changchun, 130021 China; 4 VIP Integrated Department, School and Hospital of Stomatology, Jilin University, Changchun, 130021 China

**Keywords:** Titania nanotubes, Minocycline, Control release, Schwann cells, Nerve regeneration

## Abstract

**Background:**

Minocycline has been widely used in central nervous system disease. However, the effect of minocycline on the repairing of nerve fibers around dental implants had not been previously investigated. The aim of the present study was to evaluate the possibility of using minocycline for the repairing of nerve fibers around dental implants by investigating the effect of minocycline on the proliferation of Schwann cells and secretion of neurotrophic factors nerve growth factor and glial cell line-derived neurotrophic factor in vitro.

**Methods:**

TiO_2_ nanotubes were fabricated on the surface of pure titanium via anodization at the voltage of 20, 30, 40 and 50 V. The nanotubes structure were characterized by scanning electron microscopy and examined with an optical contact angle. Then drug loading capability and release behavior were detected in vitro. The TiO_2_ nanotubes loaded with different concentration of minocycline were used to produce conditioned media with which to treat the Schwann cells. A cell counting kit-8 assay and cell viability were both selected to study the proliferative effect of the specimens on Schwann cell. Reverse transcription-quantitative PCR and western blot analyses were used to detect the related gene/protein expression of Schwann cells.

**Results:**

The results showed that the diameter of TiO_2_ nanotubes at different voltage varied from 100 to 200 nm. The results of optical contact angle and releasing profile showed the nanotubes fabricated at the voltage of 30 V met the needs of the carrier of minocycline. In addition, the TiO_2_ nanotubes loaded with the concentration of 20 μg/mL minocycline increased Schwann cells proliferation and secretion of neurotrophic factors in vitro.

**Conclusions:**

The results suggested that the surface functionalization of TiO_2_ nanotubes with minocycline was a promising candidate biomaterial for the peripheral nerve regeneration around dental implants and has potential to be applied in improving the osseoperception of dental implant.

## Background

During the past decades, titanium and its alloys have become the key materials for dental implants, owing to their biocompatibility, chemical stability, and similar mechanical strength with human bones [1, 2]. However, the native oxide layer of titanium hardly can directly bond with bone to promote new bone formation in the early stage of osseointegration. Multiple studies have been investigated to optimize dental implants by surface modification via methods such as blasting, plasma spraying of hydroxyapatite, sandblasting and etching, and anodic oxidation. Among the methods above, it suggested that formation of a titanium implant surface with a nanostructure could reinforce osseointegration because the surface area is markedly increased and the surface topography can be nano modified to resemble native bone tissue. TiO_2_ nanotubes surfaces with the optimal length scale for cell adhesion and differentiation can induce the migration of osteoblasts and mesenchymal stem cells, and hence reinforce interactions between implant surfaces and cells [3–6].

Although lots of studies focus on improving bone regeneration around implants and osseointegration between implants and bone tissue, researches on nerve regeneration around dental implants are rare. Whereas, these nerve regeneration were important to implant osseoperception, defined as the sensation arising from mechanical stimulation of a bone-anchored prosthesis, transmitted by mechanoreceptors that may include those located in bone, joint, mucosal and periosteal tissues. Previous study indicated that there may be some reinnervation around osseointegrated implants and the regenerated nerve fiber around implants was the key factor for dental implants to respond to the biting load; however, due to the low density of nerve fiber and the lack of periodontal ligaments and mechanoreceptors, higher levels of perceptual threshold for implants than those for the natural tooth make it hard for patients to perceive the larger chewing force in time, and this kind of chewing force loaded onto the implant may cause bone resorption and finally lead to implant failure [7–9]. Therefore, the improvement of nerve regeneration around implants to enhance the sensory perception of the implant-supported denture is of clinical significance.

To some extent, the speed of axonal outgrowth determines the peripheral nerve regeneration [10]. Schwann cells derive from the neural crest and widespread in the peripheral nervous system (PNS). They can form compact myelin around large diameter axons, which are crucial for maintaining the integrity of axons. In addition, Schwann cells could proliferate and provide structural and trophic support for axonal regrowth when nerve injury occurred [11]. Numerous studies have been investigated to enhance the osseoperception of dental implant using Schwann cells [12, 13]. It was reported that a variety of neurotrophic factors secreted by Schwann cells could induce stem cells to differentiate into neuron-like cells [14]. Moreover, bioactive molecules secreted by these cells significantly contribute to the maturation and regeneration of periodontal Ruffini endings [15]. Therefore, Schwann cells were served as an in vitro cell model to investigate the enhancement of osseoperception around dental implants.

Accumulating evidence suggested that minocycline, a second generation tetracycline antibiotic, is a potential therapeutic drug for several neurodegenerative and psychiatric disorders such as neurodegenerative disorders, including cerebral ischemia, amyotrophic lateral sclerosis, Parkinson’s disease, Huntington’s disease, spinal cord injury, Alzheimer’s disease, and multiple sclerosis [16–18]. The mechanisms responsible for the pharmacological actions of minocycline are its influence on inhibition of microglial activation, attenuation of apoptosis, suppression of free-radical production, changes in leucocyte function. Although many of the benefits of minocycline are likely to be derived from its action in the central nerve system, its effects on T cells and other leucocyte subsets could also happen in the periphery [16]. In addition, minocycline also prevents glial cell proliferation and inhibits the activation of p38 MAPK [14]. In a recent study, minocycline was shown to protect Schwann cells from ischemia-like injury and promotes axonal outgrowth in bioartificial nerve grafts [19]. Meanwhile, in order to recover osseoperception for avoiding overbite, it would be highly desirable to regenerate nerve fibers around dental implants. Yet whether minocycline can improve the regeneration of nerve fibers around dental implants is still unknown.

A twin approach to mechanical design considerations is the biochemical manipulation of the local microenvironment using biomolecules that act through either endocytosis or surface interactions. TiO_2_ nanotubes have been used as drug-eluting implants capable of releasing the drug directly from its surface to prevent infection with minimum side effects [20]. To date, there has been no report on titanium surface with TiO_2_ nanotube structure containing minocycline, and effects on Schwann cells for nerve regeneration. Therefore, the objectives of this study were to: (1) develop a novel bioactive TiO_2_ nanotube surface incorporating minocycline; and (2) investigate its effects on viability, proliferation and related gene and protein expression of Schwann cells for first time. The following hypotheses were tested: (1) Different voltages would influence the surface morphology of TiO_2_ nanotubes and release behavior of minocycline; (2) TiO_2_ nanotube structure and minocycline incorporation would not harm the Schwann cells; (3) TiO_2_ nanotubes loading with minocycline would promote the proliferation and enhance related gene/protein expression of Schwann cells.

## Methods

### Preparation and characterization of TiO_2_ nanotubes

TiO_2_ nanotubes were prepared on the surface of pure titanium via anodization following previously published procedures [21, 22]. Briefly, commercial titanium foils (1 mm in thickness, 10 mm in diameter, 99.6% purity, Baoji Metal Co, Ltd, Shanxi, China) were successively sonicated in acetone, ethanol, and distilled water. After air drying, titanium foils acted as an anode and platinum as a cathode, and were inserted into 75% glycerol (Sinopharm Chemical Reagent Co, Ltd, China) solution containing 0.27 M ammonium fluoride (Beijing Chemical Works, China) under 20 V, 30 V, 40 V and 50 V (DC power supply, Pinggo WYJ 3A 60 V, Hangzhou, China), respectively for 6 h at room temperature under ultrasonicated condition (50% ultrasonic power, KQ3200DE, Kunshan, China). After anodizing, nanotube products were rinsed with water, and sequentially soaked in pure ethanol and distilled water overnight. After air drying, nanotubes were then annealed at 400 °C (5 °C/min) for 3 h and then gradually cooled. Afterwards, nanotubes structure were characterized by scanning electron microscopy (SEM, Hitachi S-520, Hitachi Ltd, Tokyo, Japan) to determine the surface morphology of the nanotubes under different voltages.

### Wettability measurement

The wettability of the titanium foils was examined with an optical contact angle (CA) measuring device (Dataphysics OCA 40 Micro; DataPhysics Instruments GmbH, Filderstadt, Germany) using 10 μL dH_2_O and 10 μL of diiodomethane at 25 °C and 45% humidity. The CA was measured using the profiles of the droplets deposited on the modified surfaces immediately after stabilization using SCA 40 software (DataPhysics Instruments GmbH) [23, 24].

### Loading efficiency and In vitro release kinetics of minocycline loaded in TiO_2_ nanotubes

Previous studies indicated that TiO_2_ nanotubes coated with bovine serum albumin (BSA) had a superior drug loading capability and release behavior [24, 25]. Therefore, in the present study, TiO_2_ nanotubes were coated with BSA following the method described previously [25–28]. In brief, TiO_2_ nanotubes were coated with BSA by immersion in BSA solution (0.5 mg/mL in PBS, Lingsheng, Shanghai, China) for 2 h and then freeze dried. After rinsing with 500 μL PBS to remove unbound BSA, BSA-coated nanotubes were further immersed in minocycline solution (100 μg/mL PBS) for 2 h and then freezing dried.

BSA-coated nanotubes were filled via a simplified lyophilization method [29, 30]. In brief, TiO_2_ nanotube surfaces (1 mm in thickness, 10 mm in diameter) were cleaned with deionized water prior to drug loading. One microliter of minocycline solution (100 mg/mL in PBS) was pipetted onto the nanotube surface and gently spread to ensure even coverage. The surfaces were then allowed to dry under vacuum at room temperature for 2 h. After drying, the loading step was repeated until 400 mg of minocycline was present in the nanotube array. Afterwards, the surfaces were rinsed quickly by pipetting 500 mL of PBS over the surface to remove any excess drug. The rinse solutions were collected and stored for further analysis.

Before the release kinetics were performed, it was important to evaluate the loading efficiency of the minocycline in the nanotubes. The concentrations of the original and the rinse solutions were measured by high performance liquid chromatography (HPLC, 2010, SHIMADZU, Japan). The loading efficiency was expressed as a percentage of loaded protein after washing. The loading efficiency was calculated by the following equation: *ŋ *= (*C*_0 _− *C*_r_)/*C*_0_ × 100%, where *ŋ* is the loading efficiency, *C*_0_ is the minocycline concentration in the original solution, and *C*_r_ is the minocycline concentration in the rinse solution [28].

To determine the effect of different titanium surfaces under different voltage on releasing behavior of minocycline, the nanotubes loaded with minocycline was immersed into 3 mL stimulated body fluid (SBF) to reach a solid/liquid volume ratio at 0.08/3 following published procedure [23]. 20 μL SBF was sampled at specific time points up to 96 h to determine minocycline concentration by high performance liquid chromatography (HPLC, 2010, SHIMADZU, Japan). At every sample taking, 20 μL fresh SBF was added back to the soaking solution. Release rate of minocycline was calculated by normalizing to the area of each film. This study was performed in triplicate for each preparation.

### Cell viability

From surface characterization and releasing curve, titanium surface with nanotubes developed at 30 V was verified to exhibit superior surface morphology, wettability and best release behavior for minocycline. Therefore, titanium foil treated at 30 V voltage was used as the substrate to determine the different concentration of minocycline influence Schwann cells viability, proliferation, apoptotic, gene and protein expression. The following groups were fabricated and evaluated in the following experiments.

1. Pure titanium control group: pure titanium foil without any modification (denoted “Pure Titanium”);
2. TiO_2_ nanotube control group: TiO_2_ nanotubes developed at 30 V loaded with 0 μg/mL minocycline(denoted “TNT groups”);
3. 5 μg/mL minocycline group: TiO_2_ nanotubes developed at 30 V loaded with 5 μg/mL minocycline (denoted “TNT + 5 MC);
4. 20 μg/mL minocycline group: TiO_2_ nanotubes developed at 30 V and delivered with20 μg/mL minocycline (denoted “TNT + 20 MC”);
5. 50 μg/mL minocycline group: TiO_2_ nanotubes developed at 30 V and delivered 50 μg/mL minocycline (denoted “TNT + 50 MC”);
6. 100 μg/mL minocycline group: TiO_2_ nanotubes developed at 30 V and delivered 100 μg/mL minocycline (denoted “TNT + 100 MC”).

All specimens were fabricated at 1 mm in thickness and 10 mm in diameter. The specimens were sterilized with ethylene oxide and degassed for 7 days for the following use.

The cell viability of Schwann cells RSC96 cells (ATCC^®^ CRL-2765™) grown on different substrates was determined by flow cytometry. The specimen for each groups were placed into a 24-well plate and then Schwann cells were seeded on the substrates at an initial density of 2 × 10^5^ cells per well. After incubation for 48 h, cells were detached from disks surfaces using 0.25% EDTA-trypsin, and fixed with 70% alcohol for 30 min at 4 °C. Cells were then washed twice with PBS, centrifuged, and incubated with Annexin-V (Dingguo, Beijing, China) 0.25 mL at 4 °C in the dark. Fifteen minutes later, propidium iodide (Dingguo) 0.5 mL was added and incubated for 5 min in the dark at 4 °C and detected by a flow cytometer (BD Pharmingen, San Diego, CA, USA) [29]. Survival rate (%) = (1 − apoptotic rate) × 100%. The experiment was performed in triplicate.

### Cell proliferation assay

Schwann cells RSC96 cells were commercially obtained from the American Type Culture Collection (ATCC^®^ CRL-2765™). The RSC96 cell line is a spontaneously immortalized rat Schwann cell line derived from long-term culture of rat primary Schwann cells [31]. Culture purity was assessed with immunofluorescence staining for Schwann cell marker protein S100 and DAPI.

Cell proliferation at 1, 4, and 7 days was carried out by Cell Counting Kit-8 assay (CCK-8; Dojindo Molecular Technologies, Inc., Kumamoto, Japan). Six specimens were made for each group for cell proliferation evaluation. Cells were cultured in Dulbecco’s Modified Eagle Medium (DMEM) containing 10% fetal bovine serum (FBS, BI, Israel) and 1% penicillin–streptomycin (Sigma-Aldrich, St. Louis, MO, USA) at 37 °C in a humidified atmosphere with 5% CO_2_. The cell proliferation test was performed using direct contact. The sterile disks for each group were transferred into 24-well plates. Schwann cells were subsequently seeded onto the surface of pure titanium, TNT group, TNT + 5MC, TNT + 20MC, TNT + 50MC, TNT + 100MC in 24-well plates at the density of 2 × 10^5^/well [32]. After culturing for 1, 4 and 7 days, the culture medium was discarded and replaced with a fresh medium. CCK-8 reagent (200 μL) was added to each well and incubated for 1 h at 37 °C. Reactions were then used for determination of absorbance at 450 nm using a microplate reader (Infinite 200 Pro, Tecan, Männedorf, Switzerland) [33]. A higher absorbance is related to a higher proliferation activity of Schwann cells on disks of different groups. Meanwhile, FITC/DAPI double staining was also used to detect the morphology of live cells on different substrates at 1 and 7 days, corresponding to the proliferation assay. All experiments were performed in triplicate.

### Gene expression

Real-time PCR assay was assessed to examine the expression of neurogenesis-related genes. The specimen for each groups were placed into a 24-well plate and then Schwann cells with a density of 2 × 10^5^ cells per well were seeded on the surface of various disks under a humidified atmosphere of 5% CO_2_ at 37 °C. After culturing for 1, 4 and 7 days, total RNA was extracted from treated cells using TRIzol reagent (Invitrogen, Carlsbad, CA, USA) and reverse transcribed into cDNA using a Revert Aid kit (Takara Bio, Otsu, Japan) according to the manufacturer’s instructions. PCR reactions were setup using SYBR Premix Ex Taq (Takara Bio) and performed with an Exicycler 96 real-time PCR system (Bioneer, Daejeon, Korea) [33, 34]. The mRNA level of cells cultured on pure titanium foils was set as the baseline. Fold changes in mRNA levels were calculated using the 2^−ΔΔCt^ method. Table 1 shows the primer sequences.

**Table 1:** Primer sequences used in the qRT-PCR Experiments

Gene	Primer sequences
GDNF	5′-CAGAGGGAAAGGTCGCAGAG-3′
	3′-ATCAGTTCCTCCTTGGTTTCGTAG-5′
NGF	5′-TCAACAGGACTCACAGGAGCA-3′
	3′-GGTCTTATCTCCAACCCACACAC-5′
GAPDH	5′-GGCACAGTCAAGGCTGAGAATG-3′
	3′-ATGGTGGTGAAGACGCCAGTA-5′

### Western blot analysis

The protein expression of GDNF and NGF was investigated by western blot analysis. The specimen for each groups were placed into a 24-well plate and then Schwann cells were seeded on the surface of disks at the density of 2 × 10^5^ cells per well. After incubation for 48 h, the protein concentration was determined using a bicinchoninic acid protein assay kit (Beyotime, Shanghai, China). Equal amounts of protein (40 μg) were resolved by SDS-PAGE electrophoresis, and then transferred to polyvinylidene fluoride film (Millipore, Bedford, MA, USA). The membranes were blocked with 5% non-fat milk in TBST (0.1% Triton X-100 in TBS) buffer for 90 min, and then incubated with primary antibodies against NGF (rabbit anti-rat, 1:1000, cat. no. WL0151, Wanleibio, Shenyang, China) and GDNF (rabbit anti-rat, 1:400, cat. no. PB0045, Boster Biological Technology, Ltd, Wuhan, China) overnight at 4 °C. Then, the membranes were incubated with horseradish peroxidase-conjugated secondary antibodies (goat anti-rabbit, 1:5000, cat. no. A0208, Biyuntian, China) and visualized with an ECL reagent (Qihai Biotech, China). The intensity of the bands, which were representative of protein levels, were assessed using Gel-Pro-Analyzer 3.0 (Media Cybernetics, Rockville, MD, USA).β-actin was used to normalize target proteins [33, 34].

### Statistical analysis

All data were checked for normal distribution with the Kolmogorov–Smirnov test. One-way analysis of variance (ANOVA) was performed to evaluate differences in surface wettability, cell viability, protein expression. Two-way ANOVA was used to assess differences in different materials and time points for cell proliferation and gene expression. Post hoc multiple comparisons were performed using Tukey’s honestly significant difference test. Statistical analyses were performed by SPSS 19.0 software (SPSS, Chicago, IL, USA) at alpha 0.05.

## Results

**Fig. 1:**
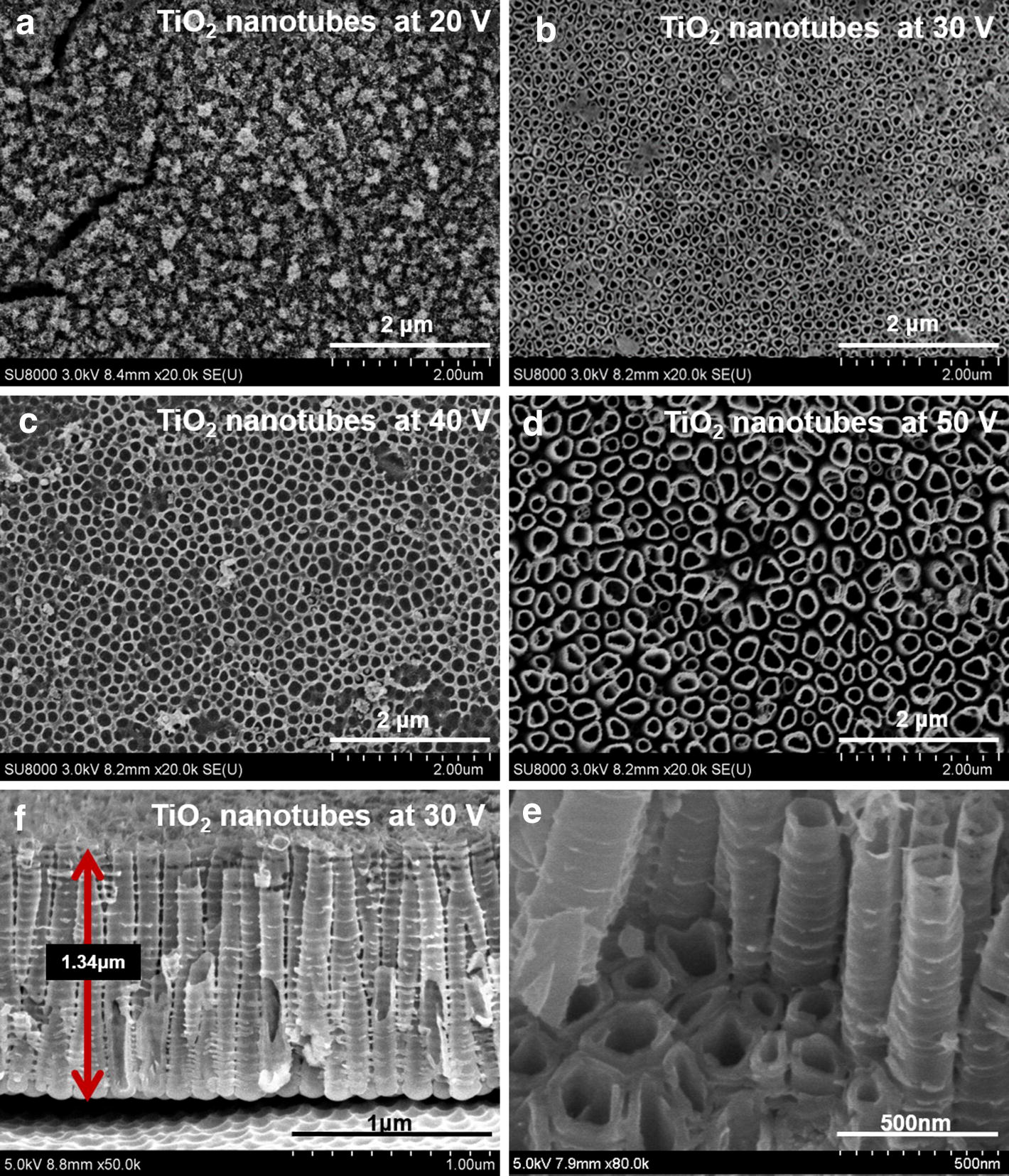
Representative surface morphology images of TiO_2_ nanotubes under different voltage: **a** 20 V, **b** 30 V, **c** 40 V and **d** 50 V. The diameters of the nanotubes fabricated under 30 V, 40 V and 50 V were, respectively (98.4 ± 11.4) nm, (136 ± 21.9) nm and (238 ± 33.5) nm, respectively. **e**, **f** Cross-sectional view of mechanically fractured sample showing that the length of the tubes was approximately 1.34 ± 0.26 µm

Figure 1 plotted representative surface morphology images of TiO_2_ nanotubes under different voltage: (a) 20 V, (b) 30 V, (c) 40 V and (d) 50 V. No nanotube structure was observed under the voltage of 20 V. While smooth nanotube structures were detected when increasing the voltage to 30, 40 and 50 V. Nanotubes formed under 30, 40 and 50 V were uniform and well-organized. Furthermore, the diameters of the nanotubes fabricated under 30, 40 and 50 V were, respectively (98.4 ± 11.4) nm, (136 ± 21.9) nm and (238 ± 33.5) nm respectively. It can be seen that the nanotubes array is uniform over the substrate with the length of 1.34 ± 0.26 µm (e, f).

**Fig. 2:**
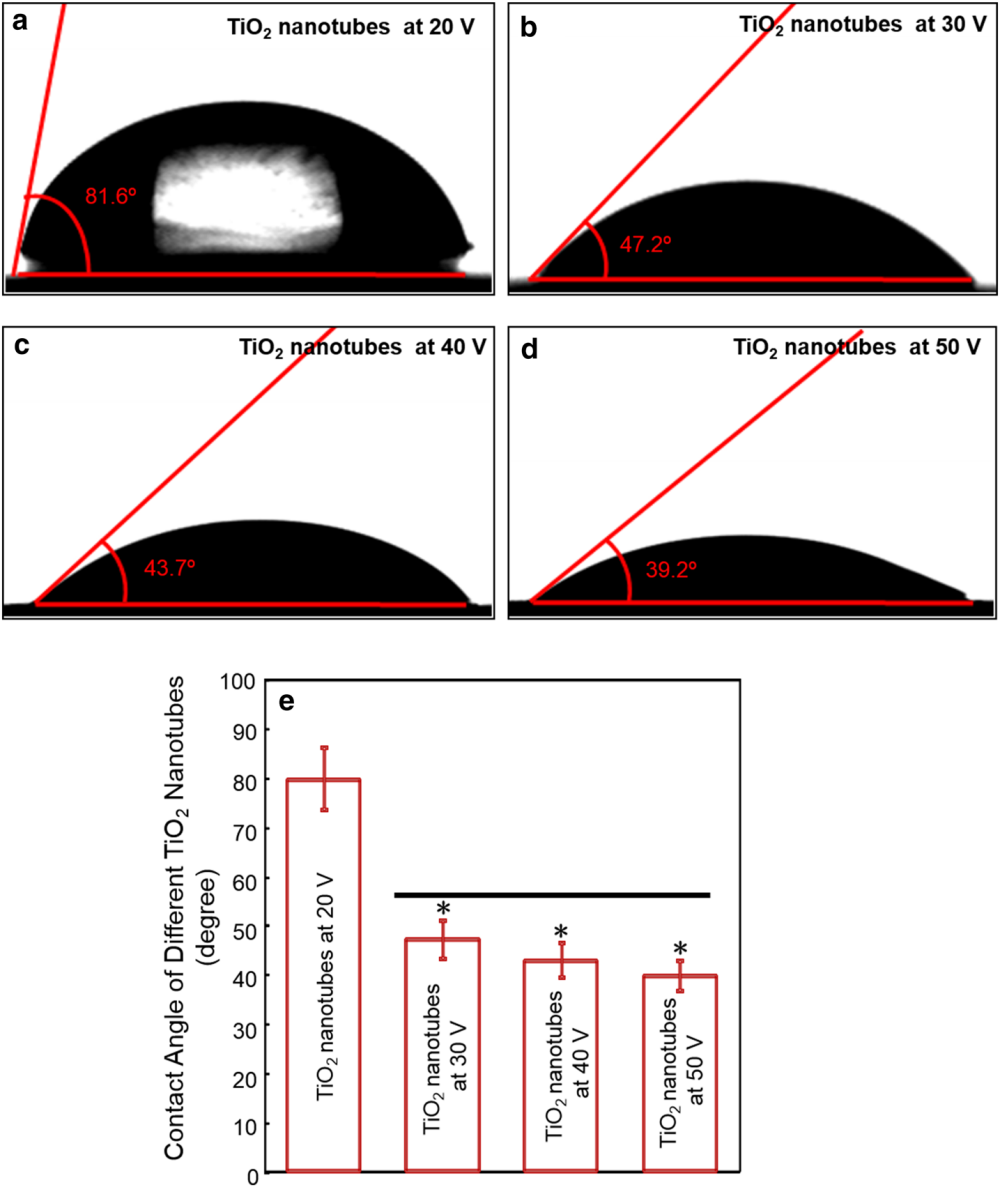
The wettability of TiO_2_ nanotubes prepared under different voltage. Contact angles as measured on **a** TiO_2_ nanotubes at 20 V, **b** TiO_2_ nanotubes at 30 V, **c** TiO_2_ nanotubes at 40 V, **d** TiO_2_ nanotubes at 50 V. **e** The contact angles for each group (mean ± SD, n = 6). Bars with dissimilar letters indicate significantly different values (p < 0.05)

Figure 2 depicted the wettability of TiO_2_ nanotubes prepared under different voltage: parts (a–d) showed the images of optical contact angle of TiO_2_ nanotubes by voltage of 20, 30, 40 and 50 V, and part (e) plotted the contact angles for each group (mean ± SD, n = 6). The data showed that titanium substrate treated under a voltage of 20 V had a significantly higher contact angle (p < 0.05), since seldom nanotube structure was formed at this condition according to Fig. 1a. The wettability was altered successfully by increasing the treating voltage. The contact angles of TiO_2_ nanotubes under 30, 40 and 50 V were, respectively (47.2 ± 11.2)°, (43.7 ± 8.4)° and (39.2 ± 6.8)° (p > 0.1).

**Fig. 3:**
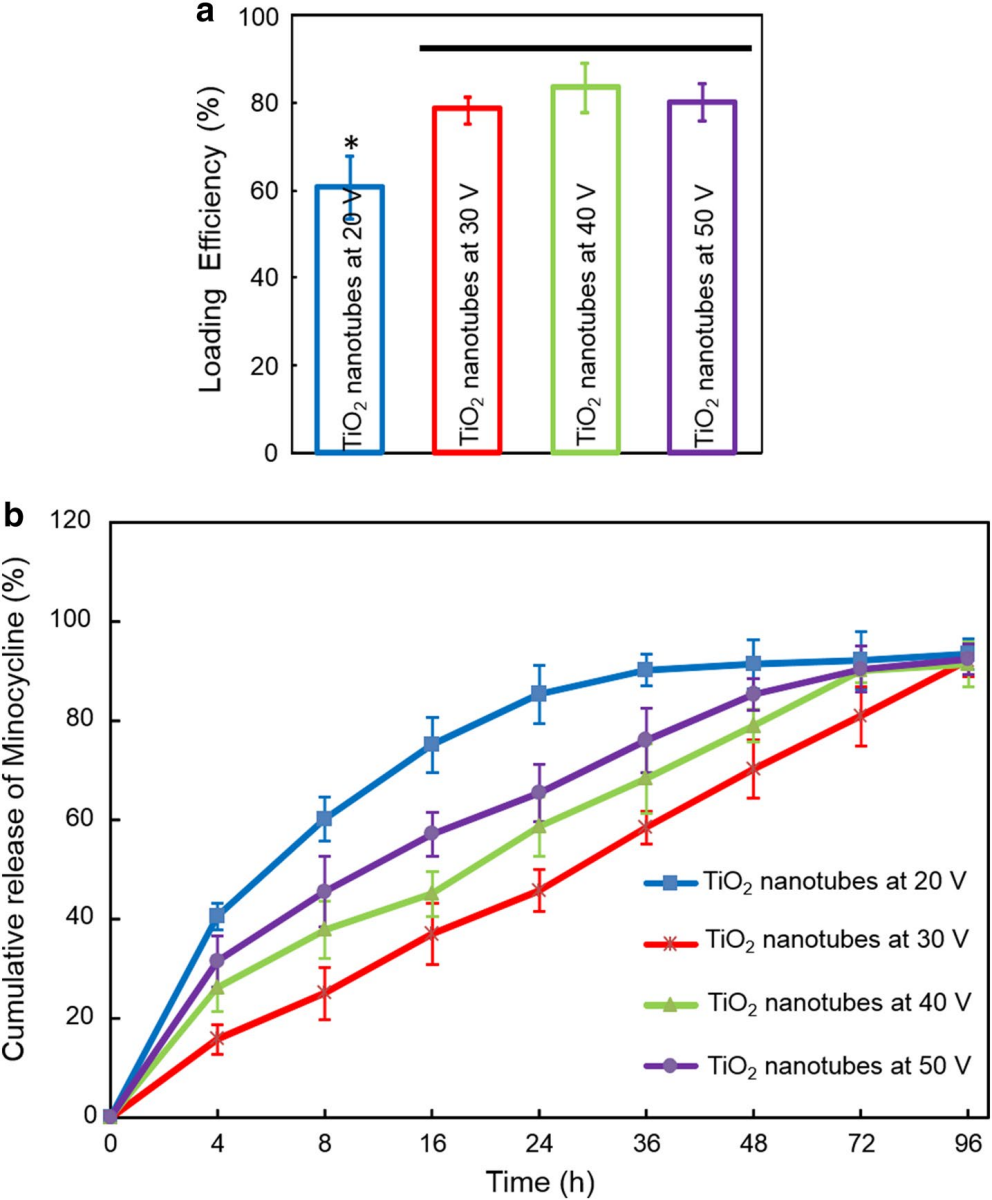
Loading efficiency and release profiles of minocycline loaded on the TiO_2_ nanotubes fabricated under different voltage. **a** The loading efficiencies of minocycline was (60.36 ± 7.15)% for TiO_2_ nanotubes developed at 20 V, (78.17 ± 3.02)% for TiO_2_ nanotubes developed at 30 V, (83.4 ± 5.63)% for TiO_2_ nanotubes developed at 40 V and (80.05 ± 4.28)% for TiO2 nanotubes developed at 50 V. Asterisk indicates significant difference value compared with other groups (p < 0.05), while horizontal line indicates values that are statistically similar (p > 0.1). **b** The initial release amount decreased obviously on the TiO_2_ nanotubes developed at 20 V, the 96 h release amount of minocycline was (93.44 ± 3.05)% for TiO_2_ nanotubes developed at 20 V, (92.19 ± 3.24)% for TiO_2_ nanotubes developed at 30 V, (91.5 ± 4.58)% for TiO_2_ nanotubes developed at 40 V and (92.53 ± 3.09)% for TiO_2_ nanotubes developed at 50 V

Figure 3a shows loading efficiencies for nanotubes loaded with 500 mg of minocycline. The results indicate approximately 75–85% of the drug is retained in the nanotubes after an initial wash. For in vitro release profiles of minocycline, the TiO_2_ nanotubes fabricated under different voltage were loaded with same amount of minocycline (100 μg/mL) to determine their release profiles in PBS at 37 °C. As illustrated in Fig. 3, the initial release amount decreased obviously on the TiO_2_ nanotubes developed at 20 V, the 96 h release amount of the loaded minocycline was (93.44 ± 3.05)% for TiO_2_ nanotubes developed at 20 V, (92.19 ± 3.24)% for TiO_2_ nanotubes developed at 30 V, (91.5 ± 4.58)% for TiO_2_ nanotubes developed at 40 V and (92.53 ± 3.09)% for TiO_2_ nanotubes developed at 50 V. The time at 80% release amount of minocycline was 20 h for TiO_2_ nanotubes developed at 20 V, 42 h for TiO_2_ nanotubes developed at 50 V, 50 h for TiO_2_ nanotubes developed at 40 V and 72 h for TiO_2_ nanotubes developed at 30 V. Minocycline in TiO_2_ nanotubes developed at 30 V had an approximately linear release profile and release obviously slower than others.

**Fig. 4:**
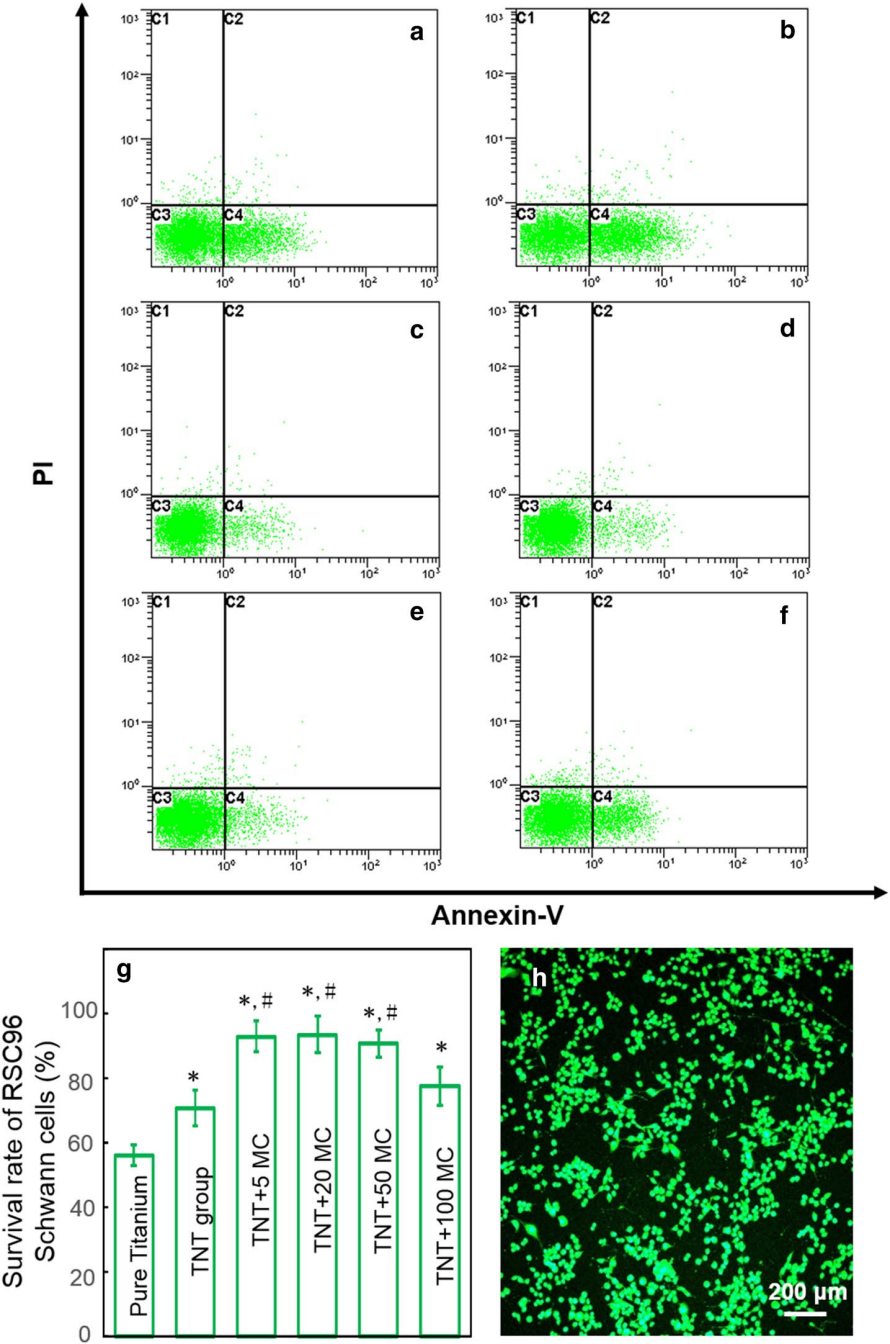
The viability of Schwann cells at different titanium substrate surface at 48 h. **a** pure titanium, **b** TNT group, **c** TNT + 5MC, **d** TNT + 20MC, **e** TNT + 50MC, and **f** TNT + 100MC; **g** the viability of Schwann cells viability at 48 h (mean ± SD, n = 6); **h** S100B staining of Schwann cells (Cells stained in green represented S100B positive cells, while the nucleus were stained in blue by DAPI). Asterisk (*) indicate significant difference values compared with pure titanium group. Hash (#) indicate significant difference values compared with TNT group (p < 0.05)

The Schwann cells viability at different titanium substrate surface at 48 h was showed in Fig. 4: parts (a–f) were the Annexin V/PI staining images by conventional flow cytometry for (a) pure titanium, (b) TNT group, (c) TNT + 5MC, (d) TNT + 20MC, (e) TNT + 50MC, and (f) TNT + 100MC; part (g) showed the viability of Schwann cells viability at 48 h; part (h) depicted the specific protein marker S100B for in Schwann cells. The cells are all positively green staining of S100B. The nanotube structure of titanium surface would significantly increase Schwann cells viability (p < 0.05). Moreover, minocycline incorporation on TiO_2_ nanotubes had a significant higher cell viability compared with pure titanium and TNT group (p < 0.05). The values of TNT + 5MC, TNT + 20MC, and TNT + 50MC are all above 90%. However, when loading concentration of minocycline reached to 100 μg/mL, the cell viability slightly decreased. The value of TNT + 100MC was not significant different with TNT group, TNT + 5MC, TNT + 20MC, and TNT + 50MC (p > 0.01).

**Fig. 5:**
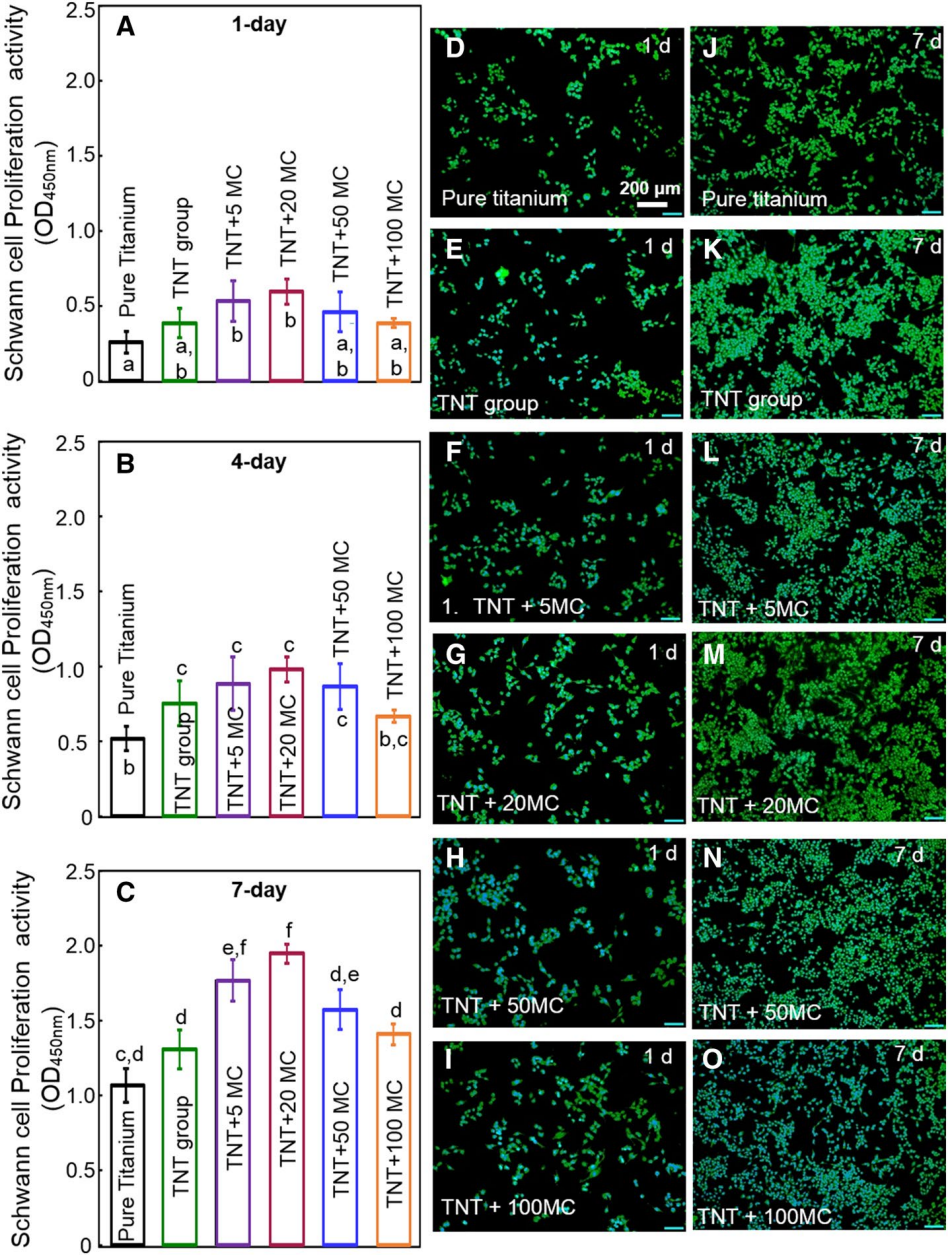
Proliferation analysis of Schwann cells subjected to titanium substrate with different concentrations of minocycline at **A** 1 day, **B** 4 days and **C** 7 days (mean ± SD, n = 6). **D**–**O** Representative fluorescence microscope images of Schwann cell stained with FITC/DAPI dual staining correspondence to the cell proliferation rate. The nanotube structure would not significantly change Schwann cells proliferation at 1 and 7 days (p > 0.1).At 4 and 7 days, The values of TiO_2_ nanotubes with minocycline incorporation would significantly higher compared with pure titanium and TNT group (p < 0.05). Values with dissimilar letters are significantly different from each other (p < 0.05)

Schwann cell proliferation rate on the surface of titanium discs with different concentrations of minocycline was plotted in Fig. 5 at (A) 1 day, (B) 4 days, and (C) 7 days. The results showed that nanotube structure would not significantly change Schwann cells proliferation at 1 and 7 days (p > 0.1). At 4 and 7 days, TiO_2_ nanotubes with minocycline incorporation would significantly enhance Schwann cells proliferation, compared with pure titanium and TNT group (p < 0.05). To be noted, TNT + 5MC and TNT + 20MC could significantly promote the proliferative rate of Schwann cells at 1, 4 and 7 days (p < 0.05). However, TiO_2_ nanotubes with high concentration of minocycline (TNT + 50MC and TNT + 100MC) had inferior proliferative rate of Schwann cells, compared with TNT + 5MC and TNT + 20MC (p < 0.05).

**Fig. 6:**
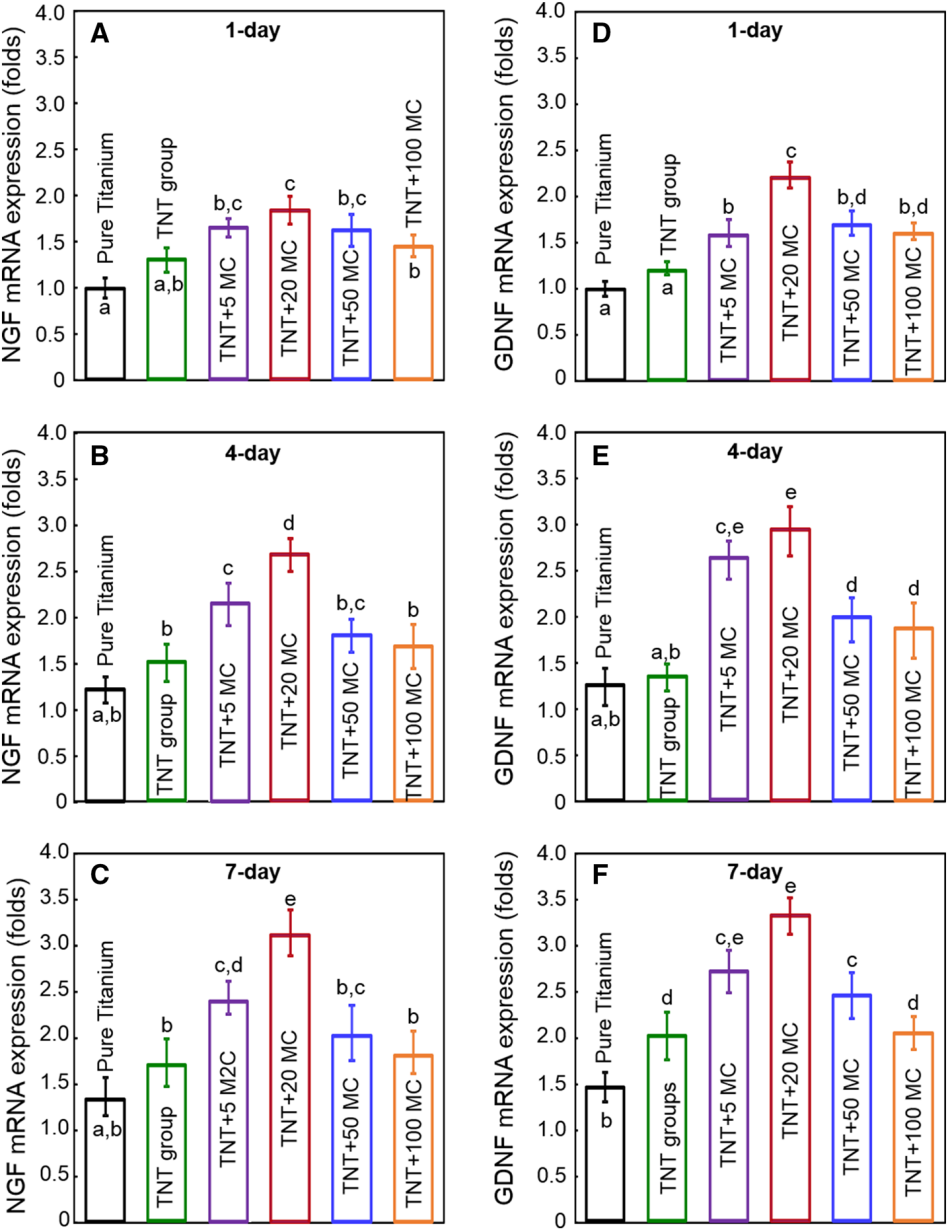
Gene expression level of (**A**–**C**) NGF and (**D**–**F**) GDNF in Schwann cells at (**A**, **D**) 1 day, (**B**, **E**) 4 days and (**C**, **F**) 7 days by real-time PCR (mean ± SD, n = 6).The mRNA expression of NGF and GNDF were up-regulated since 1 day in groups with minocycline incorporation. TNT + 20MC dramatically and continuously increased from 1 to 7 days. NGF expression in TNT + 20MC at 7 days was approximate 2.5-fold and 2-fold that of pure titanium and TNT group, respectively. GDNF expression in TNT + 20MC at 7 days was approximate over 2-fold and 1.5 fold that of pure titanium and TNT group, respectively. Values with dissimilar letters are significantly different from each other (p < 0.05)

Figure 6 plotted the mRNA levels of NGF (A–C) and GDNF (D–F) in Schwann cells seeding on the different titanium substrate at 1 day (A, D), 4 days (B, E) and 7 days (C, F). The results showed that up-regulations of NGF and GNDF were observed as early as 1 day in groups with minocycline incorporation. NGF and GDNF expression in TNT + 20MC dramatically and continuously increased from 1 to 7 days, which were over 2-fold and 1.5-fold that of 1 days. Meanwhile, NGF expression in TNT + 20MC at 7 days was approximate 2.5-fold and 2-fold that of pure titanium and TNT group, respectively. GDNF expression in TNT + 20MC at 7 days was approximate over 2-fold and 1.5 fold that of pure titanium and TNT group, respectively.

**Fig. 7:**
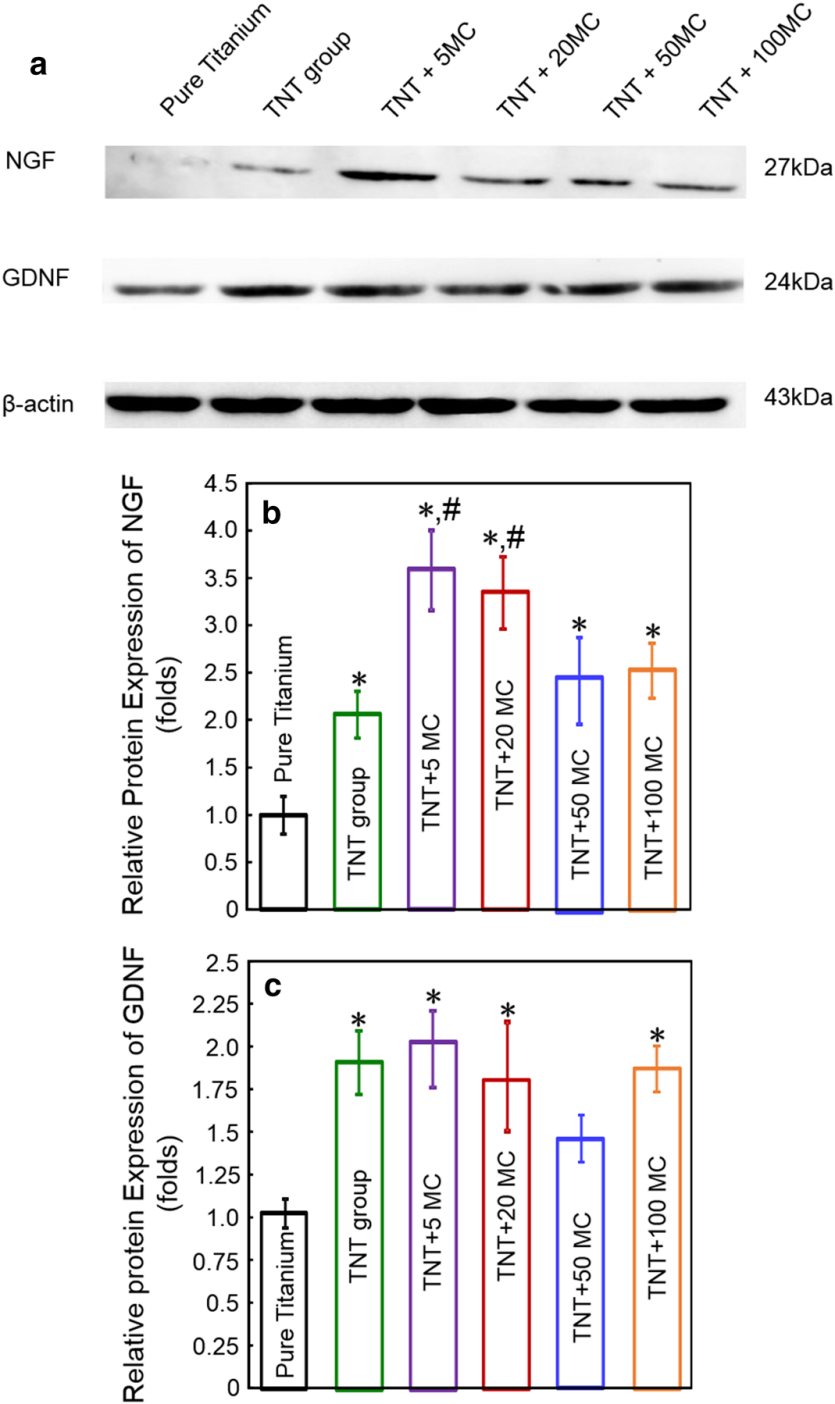
NGF and GDNF protein expression of Schwann cells seeding on different titanium substrate at 48 h (mean ± SD, n = 6). **a** Whole cell extracts were analyzed by Western blotting to determine expression levels of NGF and GDNF following seeding on different substrate with minocycline. **b**, **c** NGF and GNDF protein expression was quantified by measuring gray levels by Image J software. β-Actin was used to normalize target proteins. Asterisk (*) indicate significant difference values compared with pure titanium group. Hash (#) indicate significant difference values compared with TNT group (p < 0.05)

The effects of different specimens on neurotrophic protein secretion in Schwann cells at 48 h were illustrated in Fig. 7. NGF and GDNF protein expression significantly increased on the disks with minocycline (p < 0.05). The levels of NGF protein expression for TNT group, TNT + 5MC, TNT + 20MC, TNT + 50MC and TNT + 100MC were 2-fold, 3.3-fold, 3.5-fold, 2.4-fold, 1.9-fold that of pure titanium. The levels of GDNF protein expression for TNT group, TNT + 5MC, TNT + 20MC, TNT + 50MC and TNT + 100MC were 1.9-fold, 2.0-fold, 1.7-fold, 1.4-fold, 1.8-fold that of pure titanium.

## Discussion

This study developed a novel bioactive TiO_2_ nanotube surface incorporating minocycline, and investigated its effects on viability, proliferation and related gene and protein expression of Schwann cells for first time. The hypotheses were proven that different voltages would influence the surface morphology of TiO_2_ nanotube and release behavior of minocycline. In addition, TiO_2_ nanotube structure and minocycline incorporation would not harm the Schwann cells. Furthermore, TiO_2_ nanotubes loading with minocycline would positively correlate with the proliferation and related gene/protein expression of Schwann cells in vitro.

As for titanium anodizing, the main reactions is ① Ti ⇔ Ti^2+ ^+ 2e^−^ at the Ti/Ti oxide interface. While, ② 2H_2_O ⇔ 2O^2− ^+ 4H^+^ and ③ 2H_2_O ⇔ 2O_2_ + 4H + 4e occurred at the Ti oxide/electrolyte interface. At both interfaces, the main reaction would be: ④ Ti^2+ ^+ 2O^2− ^⇔ TiO_2_ + 2e. The mechanism of the formation of the TiO_2_ nanotubes on the surface upon voltage was probably due to the competition between Reaction ① and Reaction ②, as the formation and dissolution of titanium dioxide determines the tube length and wall thickness [20]. With the same electrolyte composition, a wide range of tube diameters can be fabricated by controlling the applied voltage on the titanium surface via anodizing process [35]. In the present study, we synthesized an anodized oxide surface of TiO_2_ nanotubes at the voltage of 30–50 V. The average tube diameter was found to increase with increasing anodizing voltage. There is a precise correlation between the anodization voltage and pore size, thus by varying the voltage substrates with different size scales can be fabricated [3, 34]. The formation of the surface not only increased the surface area and roughness, but also provided a better surface for adhesion. In order to improve the top morphology of TiO_2_ nanotubes, either a pre-treatment or a post-treatment of the substrate is applied. The normal substrate pre-treatment includes polishing or double anodization. In this study, there was no nanostructure at the voltage of 20 V probably due to insufficiency of pre-treatment or post-treatment. However, numerous studies indicated that the diameter of the nanotubes fabricated at the voltage of 20 V would be less than 100 nm [36]. It has been verified that TiO_2_ is more bioactive than pure titanium alone. Human mesenchymal stem cells on TiO_2_ nanotubes with a diameter range from 70 to 100 nm had various levels of osteogenic up-regulation and showed a significantly higher level of alkaline phosphate and osteocalcin expression than those growing on the smaller nanotube diameter [37, 38]. In addition, Jain et al. found Schwann cells on the nanofibrous substrates had a tendency to grow along the nanofibers. Therefore, morphology of Schwann cells would be affected by nanostructure more or less [29]. In the present study, the diameter of nanotubes at a voltage of 30 V was nearly 100 nm, which is potentially suitable for application in neurogenesis.

Wenzel defines the equilibrium contact angle by the equation: r(S_S _− S_SL_) = S_L_cos*θ*, where *θ* is the contact angle, S is the surface energy of solid (S) or liquid (L), and r is the roughness factor, defined as (actual surface)/(geometric surface). For hydrophilic surfaces (S_S_ > S_SL_, *θ *< 90°), an increase in the roughness factor (r) leads to a decrease in the contact angle (*θ*) [21]. Therefore, the nanotubes fabricated at the voltage of 30–50 V displayed a much higher hydrophilic surface than others probably due to a significantly increased surface area of the nanotubes.

Bovine serum albumin (BSA), which is nontoxic, biocompatible and biodegradable, played an important role in transport different drug molecules. The protein–drug interactions have been investigated in many studies, such as penicillin, sulfonamides, indole compounds, benzodiazepines and so on [39]. Apart from above, BSA was often used as a protein model due to its stability, low cost and structural homology with human serum albumin. Previous study demonstrated that drug could bind to the C–O, C–N or N–H groups of the polypeptide chain of the BSA and hydrogen bond may be formed between the drug and the BSA [27, 40]. Furthermore, there would be stable combination between minocycline and BSA, as minocycline carried a positive charge while BSA is a large molecule with a net negative charge at a neutral pH environment. Therefore, BSA was selected as a drug carrier in the present study. In addition, the surfaces of most metal oxide films are inherently charged as a consequence of the equilibration of charged crystalline lattice defects within the surface. Depending on the net concentration of lattice defects the surface may be positively or negative charged. The surface of TiO_2_ nanotubes consisted of terminal hydroxyl groups, which results in a small negative charge on the surface [28]. At the same time, minocycline carried a positive charge lead to a stable combination relative that would be benefit to the releasing of minocycline.

The large surface area of the nanotube structure and the ability to precisely tune pore size, wall thickness, and nanotube length to optimize biotemplating properties along with their surface characteristics were among the many desirable properties to use these types of surfaces as drug-eluting coatings for implantable devices [3, 30]. Therefore, by changing the nanotube diameter, wall thickness, and length, the release kinetics can be altered for each specific drug to achieve a sustained release [28, 30]. Previous studies demonstrated the nanotube surfaces could exhibit very hydrophilic behavior as the diameter varied from 12 nm to 180 nm and the length varied from 200 to 360 nm [28]. Peng et al. found that elution kinetics of paclitaxel and BSA were influences by nanotubes diameter, with nanotubes of 100 nm of diameter releasing the most drug for up to 3 weeks [41]. As expected, this study fabricated TiO_2_ nanotubes with the diameter of nearly 100 nm at the voltage 30 V in order to get a slower and sustained release from the nanotubes.

Cell lines are indispensable tools in biological research as they are readily available, free of genetic variations, and can be expanded without limitations [42]. Schwann cells are not an exception in this regard. Some unique features of Schwann cell lines that distinguish them from their cognate primary Schwann cells have been already revealed, such as the overexpressed platelet-derived growth factor receptors (PDGFRs) in neurofibrosarcoma-derived Schwann cell lines [31] and the significant differences in expression of mature Schwann cell markers between primary Schwann cells and Schwann cell lines [43]. However, because of tissue heterogeneity and difficulties in the isolation and culture of primary Schwann cells, many Schwann cell lines have been established and employed successfully in many studies [44, 45]. RSC96 is a spontaneously immortalized rat Schwann cell line [31]. Unlike immortalization by oncogene transfection or virus infection, spontaneous immortalization is the ability of normal diploid cells to overcome cell ageing in the absence of deliberately added exogenous agents. Therefore, RSC96 cell line was used in the present study to represent the principal glial cells of the peripheral nervous system.

Minocycline was a semisynthetic tetracycline that has been used for over 40 years. It is a small (495 Da), highly lipophilic molecule capable of crossing the blood–brain barrier. Previous study indicated that minocycline was neuroprotective in an animal model of ischaemia [46]. Since then, there have been numerous reports of the efficacy and neuroprotective effects of minocycline in various models of neurological disease [16, 17]. The probably mechanisms of minocycline contribute to the activity of neuroprotectant were: inhibition of microglial activation, attenuation of apoptosis, suppression of free-radical production, or inhibition of MMPs, changes in leucocyte function [16]. Moreover, Machado et al. demonstrated that minocycline inhibits enzymatic activity of gelatin proteases activated by ischemia after experimental stroke and is likely to be selective for MMP-9 at low doses [47]. Schwann cells have been shown to play a critical and substantial role in peripheral nerve regeneration, as the proliferation of Schwann cells supported the rapid regeneration of injured peripheral nerves by providing bioactive substrates needed for axonal outgrowth. Following peripheral nerve injury, Schwann cells proliferate, form a Büngner belt and devour the debris of denatured axons and myelin together with macrophages [32, 48–50]. In this study, both the cell viability and CCK-8 assay analysis indicated that minocycline promoted Schwann cells proliferation. Similar results have previously been reported, that minocycline may protect kidney epithelial cells or myocytes [51, 52]. In the present study, the increasing minocycline concentration could promote cell viability and proliferation. However, the optimal concentration of 5–20 μg/mL had the most beneficial effect on Schwann cells. This is in line with previous research that when Schwann cells were exposed to 5 μg/mL minocycline the cell death rate was a third and by application of 50 μg/mL the cell death rate was nearly a half [19].

Besides, Schwann cells also secreted a range of neurotrophic factors, including NGF and GDNF, which play neuron-protective and axon-inducing roles. It has been suggested that the proliferation and increased secretory function of Schwann cells contribute to the regeneration of neural tissue [48–50, 53]. As an endogenous neurotrophin, NGF made some contribution to trophic and differentiating activity on neurons of the central and peripheral nervous systems. Previous studies indicated that NGF might be related not only to a neuroprotective activity against apoptosis, but also to the formation of new neural pathways, since NGF had the ability to promote neural plasticity and axonal regeneration [54]. In addition, previous study showed that minocycline treatment increased the number of NGF positive cells in accordance with the result of present study that was the reason of the increasing of gene expression and protein secretory [55]. The underlying mechanism might be that minocycline could regulate the expression of TrkA, which binds to NGF with high affinity and activates the downstream PI-3K/Akt pathway to inhibit the excessive release of glutamate to reduce brain injury due to Ca^2+^ overload, that enhance cell survival and neuronal differentiation [55]. Glial cell-line derived neurotrophic factor (GDNF) belongs to the TGF-β family of neurotrophic factors and plays various and distinct roles in the neuronal signaling pathways. At the same time, GDNF has also been established as a growth factor for the survival and maintenance of dopamine neurons. In previous clinical trials, GDNF treatment in Parkinson’s disease patients led to improved motor function and GDNF has been found to be down regulated in Parkinson’s disease patients [56]. In our present study, both the mRNA and protein expression levels of GDNF were increased by low dose minocycline. Similar results have been previously reported, that GDNF expression was promoted by minocycline [57, 58]. In present study, minocycline could up regulate the key neurogenesis-related gene and protein at a low dose. Therefore, application of low dose minocycline on the surface of titanium substrate was potential beneficial for neurogenesis and osseoperception. Further studies should elucidate the related pathway by which modulates cell proliferation and secretion. Neurophysiological experiments should also be evaluated in vivo in order to investigate the effect of surface functionalization of TiO_2_ nanotubes with minocycline on nerve repairing.

## Conclusions

This present study synthesized a novel bioactive TiO_2_ nanotube surface incorporating minocycline, and investigated its effects on viability, proliferation and related gene and protein expression of Schwann cells for first time. The hypotheses were proven that different voltages would influence the surface morphology of TiO_2_ nanotubes and release behavior of minocycline. In addition, TiO_2_ nanotube structure and minocycline incorporation would not harm the Schwann cells. Furthermore, TiO_2_ nanotubes loading with minocycline would positively correlate with the proliferation and related gene/protein expression of Schwann cells in vitro. The surface functionalization of TiO_2_ nanotubes with minocycline may open a new direction for the repairing of nerve fibers around dental implants, and has potential to be applied in improving the osseoperception of implant denture.
